# MiR-373 targeting of the Rab22a oncogene suppresses tumor invasion and metastasis in ovarian cancer

**DOI:** 10.18632/oncotarget.2577

**Published:** 2014-11-07

**Authors:** Yue Zhang, Fu-Jun Zhao, Li-Lan Chen, Luo-Qiao Wang, Kenneth P. Nephew, Ying-Li Wu, Shu Zhang

**Affiliations:** ^1^ Department of Obstetrics and Gynecology, RenJi Hospital, Shanghai Jiao-Tong University School of Medicine, Shanghai Key Laboratory of Gynecologic Oncology, Shanghai, 200127, China; ^2^ Department of Urology, Shanghai First People's Hospital, Shanghai Jiao-Tong University, Shanghai, 200080, China; ^3^ Medical Sciences, Indiana University School of Medicine, Bloomington, IN 47405, US; ^4^ Department of Pathophysiology, Chemical Biology Division of Shanghai Universities E-Institutes, Key Laboratory of Cell Differentiation and Apoptosis of National Ministry of Education, Shanghai Jiao-Tong University School of Medicine, Shanghai, 200025, China

**Keywords:** ovarian cancer, miR-373, Rab22a, invasion, metastasis

## Abstract

Metastasis is major cause of mortality in patients with ovarian cancer. MiR-373 has been shown to play pivotal roles in tumorigenesis and metastasis; however, a role for miR-373 in ovarian cancer has not been investigated. In this study, we show that the miR-373 expression is down-regulated in human epithelial ovarian cancer (EOC) and inversely correlated with clinical stage and histological grade. Ectopic overexpression of miR-373 in human EOC cells suppressed cell invasion *in vitro* and metastasis *in vivo*, and the epithelial–mesenchymal transition process. Silencing the expression of miR-373 resulted in an increased migration and invasion of EOC cells. Using integrated bioinformatics analysis, gene expression arrays, and luciferase assay, we identified Rab22a as a direct and functional target of miR-373 in EOC cells. Expression levels of miR-373 were inversely correlated with Rab22a protein levels in human EOC tissues. Rab22a knockdown inhibited invasion and migration of EOC cells, increased E-cadherin expression, and suppressed the expression of N-cadherin. Moreover, overexpression of Rab22a abrogated miR-373-induced invasion and migration of EOC cells. Taken together, these results demonstrate that miR-373 suppresses EOC invasion and metastasis by directly targeting Rab22a gene, a new potential therapeutic target in EOC.

## INTRODUCTION

Ovarian cancer is the leading cause of cancer deaths from gynecological malignancy in Western countries. According to the American Cancer Society, in 2013, 22,240 new cases of ovarian cancer and 14,230 deaths due to ovarian cancer were reported in the US. Despite great advances in chemotherapy and surgical treatment of this disease, 70 to 90% of women with ovarian cancer develop relapse or metastasis, and the 5-year survival rate of patients with advanced ovarian cancer who have peritoneal metastasis remains at approximately 30% [1]. The overall prognosis for patients with ovarian cancer is a consequence of aggressive metastatic behavior. Ovarian cancer is more likely to metastasize via intraperitoneal (i.p.) dissemination than via hematogenous or lymphatic routes. Factors involved in the pathogenesis of ovarian cancer metastasis include metastasis suppressor genes (*Nm-23*, *Kiss-1*, *KAI1*, *E-cadherin*, *BRMS1*), epithelial-mesenchymal transition (EMT), tumor microenvironment, chemokines, and anoikis resistance have been identified [2–7]. In addition to those signaling molecules and cytokines, recent findings have shown that non-protein-coding RNAs, especially microRNAs (miRNAs) are often deregulated in ovarian cancer and are involved in the tumorigenesis and progression of ovarian cancer [8]. The miRNAs are endogenous 19–25 nt noncoding RNAs that can bind the 3′-untranslated region (3′-UTR) of specific genes to inhibit the translation of target gene, and an individual miRNA can target up to 200 target transcripts. While the number of known human miRNAs is continuously increasing, the roles of most of these miRNAs in human physiological and pathological processes remain to be elucidated. The miRNAs are usually dysregulated and function either as tumor suppressors or oncogenes in the initiation and progression of human carcinomas, including ovarian cancer [9]. Few miRNAs, such as miR-200 family [10, 11], let-7 family [11], miR-21 [12] and miR-214 [13] have been studied for their roles in ovarian cancer carcinogenesis.

Previously, we examined miRNA expression profiles between ovarian cancer cells and the stem-like ovarian cancer-initiating cells (OCICs) [14] and identified deregulated miRNAs highly related to the metastatic capability of OCICs (our unpublished observations). Among those, miR-373 was frequently down-regulated in OCICs and ovarian cancer cells, although the role of miR-373 in ovarian cancer was not clear. In this study, we investigated the potential tumor suppressor effect of miR-373 in ovarian cancer. We identified a direct target of miR-373, Rab22a, which is a member of the Rab family of small GTPases. We show for the first time that the miR-373/Rab22a axis contributes to migration and invasion in ovarian cancer and may represent a potential therapeutic target for the disease.

## RESULTS

### MiR-373 is down-regulated in ovarian cancer tissues and cell lines and associated with tumor invasion and metastasis

**Figure 1:**
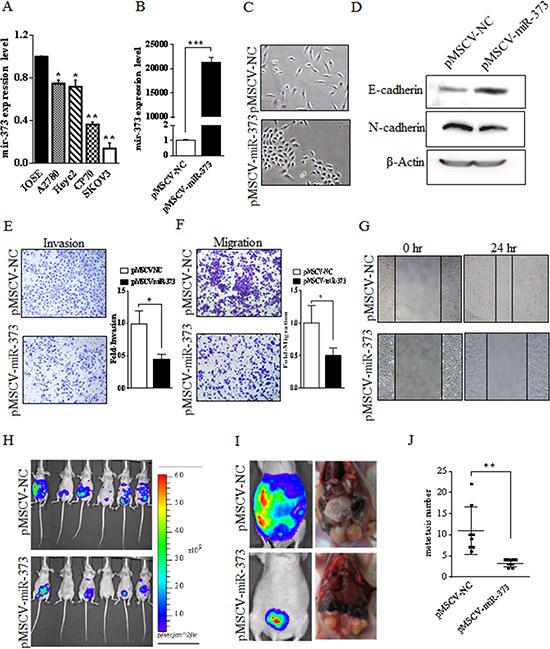
miR-373 is frequently down-regulated in ovarian cancer cell lines and miR-373 inhibits the invasion and metastasis of SKOV3 cells *in vitro* and *in vivo* **(A)** Compared to the IOSE cells, the expression of miR-373 was significantly down-regulated in all four EOC cell lines. The expression of miR-373 was the lowest in SKOV3 cells. EOC, epithelial ovarian cancer; IOSE, immortalized nontumorigenic human ovarian surface epithelial; ^*^p < 0.05 and ^**^p < 0.01. **(B)** SKOV3 cells express high level of miR-373 after transfection with pMSCV-miR-373 compared to the mock control, ^***^p < 0.001. **(C)** The effect of miR-373 on morphological changes of SKOV3 cells. More rounded epithelial-like morphology was observed in the miR-373-transfected-SKOV3 cells (bottom), while spindle-shaped mesenchymal-like morphology was observed in the mock control cells (top). **(D)** E-cadherin (epithelial marker) augmentation and N-cadherin (mesenchymal marker) reduction in SKOV3 after the expression of miR-373. **(E-F)** Transwell invasion and migration assays of SKOV3 cells expressing miR-373 or mock control. Representative images (×100) are shown on the left and the quantification of five randomly selected fields is shown on the right. The invasive and migratory potential of miR-373*-*expressing cells showed a strong reduction compared with mock controls. **(G)** Decreased cellular migration in miR-373-transfected SKOV3 cells was confirmed by wound scratch assay. **(H-I)** Representative bioluminescent images of disseminated tumor in nude mice i.p. injected with SKOV3^luc^-pMSCV-miR-373 cells (bottom) and SKOV3^luc^-pMSCV-NC cells (top), respectively. **(J)** The anatomical location of metastatic tumors upon macroscopic examination reflected bioluminescence and the number of metastatic tumor between two groups was significantly different, ^**^p < 0.01.

Previous studies have indicated that miR-373 was frequently down-regulated in colon cancer [15] and hilar cholangiocarcinoma [16]. To explore the expression and significance of miR-373 in ovarian cancer carcinogenesis, the expression of miR-373 was detected in 30 primary epithelial ovarian cancer (PEOC) samples and 15 benign epithelial ovarian tumor tissues using TaqMan quantitative RT-PCR analysis. The expression of miR-373 was down-regulated (*P* = 0.0104) in PEOC compared to benign tumor (Table 1). The expression of miR-373 was remarkably lower (*P* = 0.0124) in advanced tumor stages (III, IV) in which lymph node or distant metastases were present compared to earlier tumor stages; furthermore, differences (*P* < 0.05) in miR-373 expression among histological differentiation (G1 vs. G2-3) were observed (Table 1). These results suggested a possible association between down-regulation of miR-373 and metastastic EOC. In addition to PEOC, miR-373 expression was detected in ovarian immortal cell line IOSE and a panel of ovarian cancer cell lines cells (Fig. 1A). Compared to the IOSE cells, the expression of miR-373 was significantly down-regulated in A2780, CP70, HeyC2, and SKOV3 cell lines. Furthermore, among these EOC cells, the lowest endogenous expression of miR-373 was observed in SKOV3 cells and the highest expression of miR-373 was observed in A2780 cells (Fig. 1A). Given the above results, we decided to use the SKOV3 and A2780 cells for the below experiments.

**Table 1:** Clinicopathological features of ovarian tissue with regard to the relative expression of miR-373

Clincal characteristic	N	miR-373 expression	*P* value
**Benign**	15	0.9209 ± 0.2135	0.0104
**Malignant**	30	0.2444 ± 0.05615	
**Age**			
<50	13	0.1984 ± 0.06737	0.8080
>50	17	0.2252 ± 0.08398	
**FIGO stage**			
I-II	8	0.2316 ± 0.06109	0.0124
III-IV	22	0.05472 ± 0.01749	
**Histology**			
G1	20	0.2249 ± 0.06201	0.0474
G2-G3	10	0.09251 ± 0.03231	

### Ectopic overexpression of miR-373 suppresses EOC cell metastasis and invasion *in vitro* and *in vivo*

To better understand the impact of miR-373 on EOC cells, a lentivirus vector expressing miR-373 (pMSCV-miR-373) was constructed and SKOV3-transfected cells were established. As shown in Fig.1B, SKOV3 cells expressed high level of miR-373 after transfection with pMSCV-miR-373 compared to the mock control (*P* < 0.001). After stable transfection, miR-373-expressing SKOV3 cells displayed a change from spindle-shaped mesenchymal-like morphology to more rounded epithelial-like morphology (Fig. 1C). No morphological changes were observed in cells transfected with the negative control. Concurrent with the morphological changes, levels of E-cadherin were higher while levels of N-cadherin were lower (*P* < 0.01) in miR-373-transfected cells relative to cells transfected with the negative control (Fig. 1D), suggesting that the overexpression of miR-373 was associated with EMT and EOC cell metastasis. To further examine this possibility, an *in vitro* cell invasion assay was performed and the number of cells migrating through the Matrigel® matrix was counted. The invasive capacity of SKOV3-miR-373 cells was reduced (*P* < 0.01) compared to vector-only cells (Fig. 1E) and the ability of miR-373-transfected cells to invade was suppressed (55%) compared with vector-only cells. Boyden chamber assays without Matrigel further demonstrated that expression of miR-373 reduced (*P* < 0.01) migration of SKOV3 cells when compared with vector-only cells (Fig. 1F). In addition, result of the wound scratch assay showed reduced migration capability of miR-373-transfected cells by approximately 49% (Fig. 1G). However, ectopic expression of miR-373 had no effect on SKOV3 cell proliferation and colony formation *in vitro* (data not shown). Taken together, these results suggest that miR-373 is a negative metastatic regulator for EOC.

To evaluate the role of miR-373 in tumor invasion and metastasis *in vivo*, disseminated ovarian cancer was generated by injecting female nude mice with human SKOV3^luc^-pMSCV-miR-373 cells i.p. for the therapeutic group, while SKOV3^luc^-pMSCV-NC cells for the control group. A panel of representative images is shown in Fig. 1H-I. At 5 weeks of i.p. injection, miR-373-transfected SKOV3^luc^ cells formed fewer metastasis to organs in the peritoneal cavity compared to the control group, as the number of metastatic tumors in the pMSCV-miR-373 therapeutic group was 3.25 ± 0.31 and 11.0 ± 1.98 in the pMSCV-NC control group (*P* = 0.005) (Fig. 1J). The SKOV3^luc^-pMSCV-miR-373-injected mice showed fewer incidence of metastasis in distant organ sites, whereas SKOV3-pMSCV-NC-injected mice showed metastatic deposits in the peritoneal wall, small intestine, colon, stomach, liver and diaphragm.

### miR-373 directly regulated Rab22a activity

**Figure 2:**
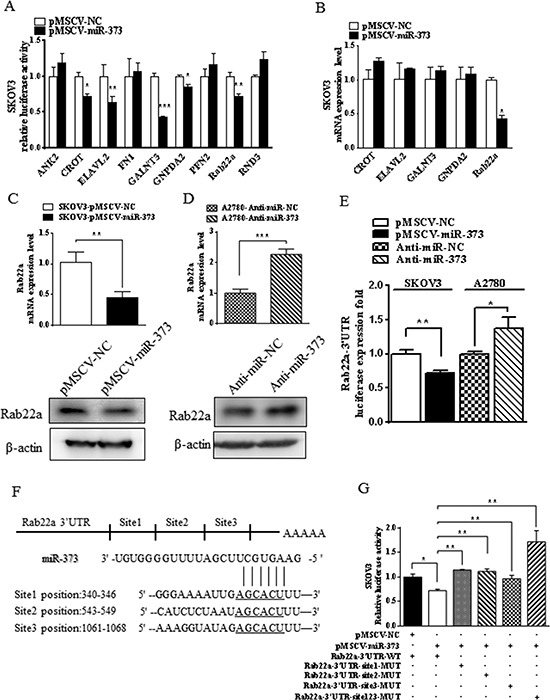
miR-373 directly regulates Rab22a in SKOV3 cells **(A)** Luciferase reporter assay was used to reveal 3′-UTRs of five candidate miR-373 targets (CROT, ELAVL2, GALNT3, GNPDA2, and Rab22a). **(B)** Quantitative RT-PCR was used to validate that only Rab22a was down-regulated in miR-373-transfected cells out of these five candidate genes. ^*^p < 0.05, ^**^p < 0.01 and ^***^p < 0.001. **(C-D)** Quantitative RT-PCR and Western blot analyses were performed to examine the effects of over- and under-expressed miR-373 on Rab22a gene expression in SKOV3 and A2780 cells, respectively. **(E)** Relative luciferase activity of Rab22a 3′-UTR in SKOV3 cells stable with or without overexpression of miR-373 and in A2780 cells transfected with anti-miR-373 inhibitor or negative control. **(F)** Putative miR-373 binding sequence in the 3′-UTR of Rab22a mRNA. **(G)** Relative activity of the luciferase gene fused with the wild-type or mutant 3′-UTR of Rab22a. ^*^P < 0.05, ^**^P < 0.01.

To investigate the potential target gene which miR-373 could regulate in ovarian cancer cells, microarray gene expression profiling of SKOV3-pMSCV-miR-373 cells and SKOV3-pMSCV-NC cells was performed. The results in two independent experiments showed that 262 transcripts, including 168 downregulated transcripts and 94 upregulated transcripts, were impacted by overexpression of exogenous miR-373 in SKOV3 cells (Supple Table 1). Data suggested that miR-373 acts as a tumor suppressor in EOC, therefore a set of 168 downregulated transcripts, which are potential direct targets of miR-373, was focused on for further experiments. Three bioinformatics-based prediction analysis softwares (PicTar, TargetScan, and miRanda) were used to identify the potential miR-373 targets. Of the 12 downregulated transcripts and potential targets of miR-373 (ANK2, CD44, CROT, ELAVL2, FN1, GALNT3, GNPDA2, PFN2, Rab22a, RND3, TGFBR1, and TGFBR2), CD44 [17], TGFBR1 [18], and TGFBR2 [19] have been reported as the direct miR-373 targets. To validate the other nine candidates, dual luciferase reporter assays were performed using constructs in which these targeting sites were cloned into the 3′-UTR of the renilla luciferase reporter gene (psiCHECK™-2). Transfection of cells with pMSCV-miR-373, resulted in reduced (*P* < 0.05) luciferase activities of CROT, ELAVL2, GALNT3, GNPDA2, and Rab22a compared to controls (Fig. 2A). Additional examination of these five genes using quantitative RT-PCR analysis showed that only Rab22a was downregulated in SKOV3-miR-373-transfected cells (Fig. 2B).

To further examine Rab22a as a direct target gene of miR-373, SKOV3 and A2780 cells were transfected with pMSCV-miR-373 and anti-miR-373 inhibitor, respectively. Both mRNA and protein expression of Rab22a were substantially decreased after ectopic miR-373 transfection in SKOV3 cells (Fig. 2C). While diverse phenomena were observed, the Rab22a mRNA and protein levels were down-regulated by anti-miR-373 inhibitor in A2780 cells (Fig. 2D). Dual luciferase reporter assays confirmed that the luciferase activities of Rab22a 3′-UTR were significantly reduced in SKOV3 cells stably transfected with pMSCV-miR-373 and the luciferase activities increased in A2780 cells transfected with anti-miR-373 inhibitor (Fig. 2E). Based on the bioinformatics prediction analysis, three miR-373-targeting sequences were identified in the 3′-UTR of Rab22a mRNA (Fig. 2F), and mutant vectors of Rab22a 3′-UTR containing four mutated bases on the predicted binding sites were constructed. Transient transfection of SKOV3 cells with the above wild-type or mutant vectors and miR-373 or mock control resulted in partial rescue of the inhibition (Fig. 2G), further supporting that Rab22a is a direct target of miR-373.

### Rab22a is down-regulated in EOC tissues and promotes tumor cells invasion and metastasis

**Figure 3:**
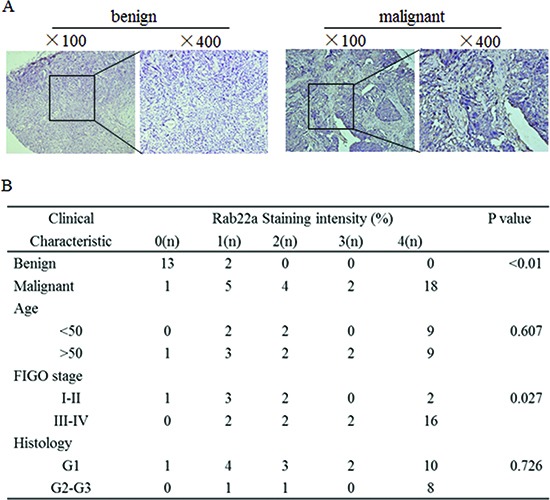
Rab22a is often overexpressed in human EOCs **(A)** Representative images of IHC staining for Rab22a in benign ovarian tumor and EOC tissues (×100 and ×400). **(B)** Clinicopathological features of ovarian tissue with regard to the relative expression of Rab22a. IHC = immunohistochemistry.

Previous reports indicated that the expression of Rab22a is increased in different cancer types, including liver cancer [20] and malignant melanoma [21], suggesting an oncogenic role for Rab22a. However, such a role for Rab22a in human EOC cells has not been investigated. IHC analysis was used to measure the expression of Rab22a protein in 30 cases of EOC tissue, 15 cases of benign ovarian tumor tissues and 10 cases of normal ovarian tissues. The expression of Rab22a protein was significantly up-regulated in EOC tissues when compared with those in benign tumor and normal ovarian tissues (Fig. 3A). Increased expression of Rab22a was observed in advanced tumor stages (III, IV), indicating that expression of Rab22a was positively correlated with FIGO stage (*P* = 0.027). No significant differences were observed based on patients' age (<50 years vs. >50 years) and histology (Fig. 3B). The overexpression of Rab22a in EOC tissues was strongly correlated with reduced expression of miR-373, suggesting that the increased expression of Rab22a might result from down-regulation of miR-373 in human EOC.

**Figure 4:**
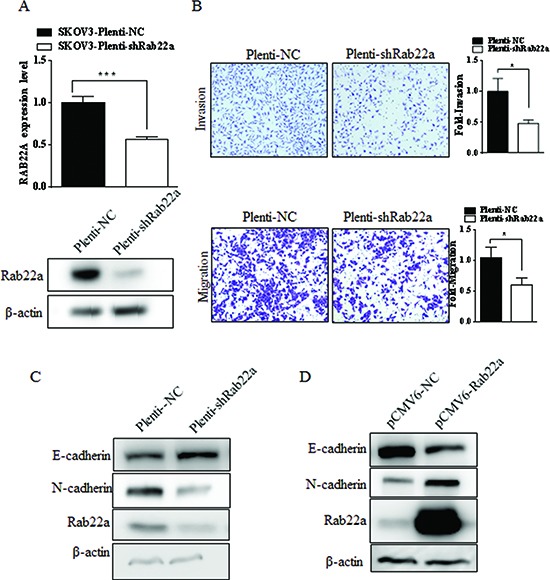
Rab22a can significantly promote migration and invasion of EOC cells **(A)** Quantitative RT-PCR and Western blot analyses were performed to examine depressing the expression of Rab22a in SKOV3 cells after stably transfected with pLenti-shRab22a. **(B)** Transwell invasion and migration assays of SKOV3 cells transfected with Rab22a-siRNA or negative control were performed to investigate the effects of Rab22a on migration and invasion of EOC cells. **(C-D)** Western blot analysis was used to assay the expression level of E-cadherin and N-cadherin in SKOV3 cells after transfection with Rab22a-siRNA or pCMV6-Rab22a, ^*^P < 0.05, ^**^P < 0.01.

To explore the biological functions of Rab22a in ovarian cancer cells, endogenous expression of Rab22a was knocked down in SKOV3 cells with specific siRNA (pLenti-shRab22a) (Fig. 4A). Rab22a knockdown was associated with reduced (*P* < 0.05) cell migration and invasion (Fig. 4B), increased expression of epithelial marker E-cadherin and decreased expression of mesenchymal marker N-cadherin (Fig. 4C). Ectopic overexpression of Rab22a in SKOV3 cells reversed these molecular changes, resulting in down-regulation of E-cadherin and up-regulation of N-cadherin (Fig. 4D).

### MiR-373 directly targets Rab22a to suppress EMT-mediated migration and invasion in EOC

**Figure 5:**
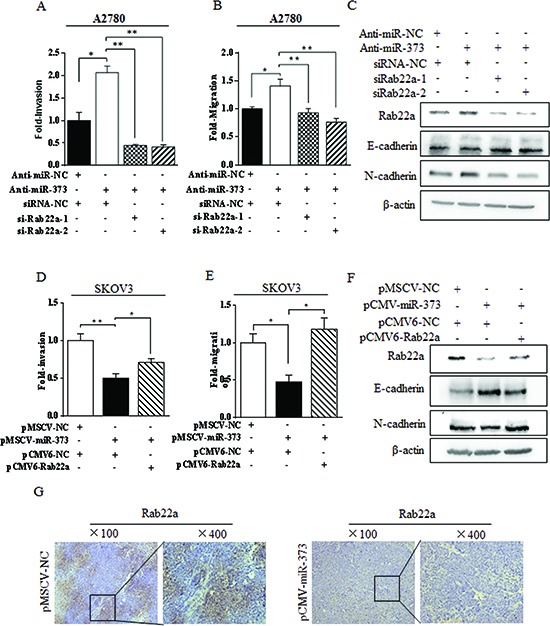
miR-373 suppressed EMT-related migration and invasion via directly targeting Rab22a in ovarian cancer cells **(A-B)** Transwell invasion assays and migration assays for A2780 cells after co-transfected with anti-miR-373 inhibitor and siRab22a. **(C)** Immunoblotting of E-cadherin and N-cadherin in A2780 cells co-transfected with anti-miR-373 inhibitor and siRab22a. **(D-E)** Transwell invasion assays and migration assays for SKOV3 cells after co-transfected with miR-373 and Rab22a. **(F)** Immunoblotting of E-cadherin and N-cadherin in SKOV3 cells co-transfected with miR-373 and Rab22a, ^*^P < 0.05, ^**^P < 0.01. **(G)** The down-regulation of Rab22a was strongly correlated with the over-expression of miR-373 in ovarian carcinoma metastatic models constructed in nude mice by SKOV3^luc^-pMSCV-miR-373 cells.

To examine a possible role for Rab22a in miR-373-mediated suppression of EOC migration and invasion, specific siRNAs against Rab22a were used. As siRab22a-2 inhibited Rab22a protein more efficiently than siRab22a-1 (Supple Fig.1), co-transfection experiments using siRab22a-1 or siRab22a-2 and anti–miR-373 were carried out. Inhibition of miR-373 expression was unable to increase cell invasion and migration in Rab22a-depleted A2780 cells (Fig. 5A-B), and western blot analysis showed that E-cadherin was positively regulated and N-cadherin was negatively regulated in A2780 cells co-transfected with anti-miR-373 and siRab22a (Fig. 5C). On the other hand, overexpression of Rab22a abrogated (*P* < 0.05) miR-373-mediated invasion and migration of SKOV3 cells (Fig. 5D-E), and E-cadherin was negatively regulated and N-cadherin was positively regulated in SKOV3 cells co-transfected with pCMV-miR-373 and pCMV6-Rab22a (Fig. 5F). The down-regulation of Rab22a was strongly correlated with the over-expression of miR-373 in xenograft ovarian carcinoma metastatic models in nude mice by SKOV3^luc^-pMSCV-miR-373 cells (Fig. 5G). Taken together, these results support the hypothesis that miR-373, by targeting Rab22a, regulates EOC migration and invasion.

## DISCUSSION

Recent studies have shown that miRNAs play a fundamental role in the invasion and metastasis of malignant cancers [22], including EOC [8]. Previous studies identified miR-373 as an oncogene that is up-regulated in a number of different human tumor types, including medullary thyroid carcinoma [23], hepatocellular carcinoma [24, 25], prostate cancer [26], gastric cancer [27], and testicular germ cell tumors [28]. However, in breast cancer miR-373 appears to have tumor suppressor activity, as re-expression of miR-373 inhibited TGF-β-induced invasion in MDA-MB-231 and MCF10A cells as well as vascular intravasation in ER^−^ breast cancer *in vivo* [19]. Chen et al. reported that down-regulation of miR-373 in hilar cholangiocarcinoma is associated with poor cell differentiation, advanced clinical stage, and shorter survival [15]. However, little is known about the role and underlying molecular mechanism of miR-373 in EOC, therefore the present study represents the first report on miR-373 in a panel of EOC cell lines and patient ovarian cancer tissues. Moreover, re-expression of miR-373 in ovarian cancer cells results in decreased cellular migration and invasion *in vitro* and *in vivo*. Taken together, these data suggest that miR-373 acts as a tumor suppressor in EOC, which is in agreement with those previous findings on this miR in breast cancer. MiR-373 belongs to miRNAs-371-373 clusters, located at chromosome 19q13.42 and matured from pri-miRNAs-371-373. Recent studies have shown that rearrangements of chromosomal band 19q13.4 represent a frequent clonal cytogenetic deviation in thyroid adenomas [29] and prostate cancer [30]. MiR-373 is located in close proximity to these chromosomal rearrangements and several groups recently have linked the expression of miR-373 with the genomic alteration [30]. Tanaka [16] and Chen [15] et al. reported that the expression of miR-373 is down-regulated by aberrant methylation in colon cancer and hilar cholangiocarcinoma, and our future studies will examine the possible mechanism(s) regulating miR-373 expression in EOC.

Our current study identified that Rab22a was a direct target of miR-373 in EOC. Down-regulation of miR-373 in EOC cells resulted in increased expression of Rab22a, resulting in enhanced EOC migration and invasion. Transient expression of miR-373 and siRNA-based knockdown of Rab22a showed essentially similar effects in ovarian cancer cells, further confirming that direct targeting of Rab22a by miR-337 plays an important inhibitory role in migration and invasion of ovarian cancer cells. Rab22a, a small GTPase, is a member of the Rab family endocytic pathway [31]. Previous reports showed that Rab22 recruits Rabex-5 (a guanine nucleotide exchange factor (GEF) for activation of Rab5) on early endosomes and activates Rab5, establishing a Rab22–Rabex-5–Rab5 cascade that is functionally important for the endocytosis and degradation of epidermal growth factor. Rab5 was reported to play key roles in the migration of cancer cells through the integrin-mediated signaling pathway [32–34]. Recently, Hu et al. found activation of Rab5 could promote TGF-β signaling [35] and it is increasingly apparent that TGF-β and TGF-β-related proteins have emerged as major inducers of epithelial-mesenchymal transition process in development and cancer. These findings shed new light on the model in which miR-373 may suppress TGF-β signaling through Rab22–Rabex-5–Rab5 cascade. Overexpression of Rab22a has been observed previously in liver cancer [20] and MM cell lines [21], suggesting an oncogenic role for Rab22a in tumorigenesis. We demonstrate that Rab22a is significantly up-regulated in human EOC tissues and overexpression of Rab22a in EOC cells may result from down-regulation of miR-373. Enforced over-expression of miR-373 down-regulate Rab22a expression in EOC cells, while Rab22a knockdown reduce EOC cells migration and invasion. Furthermore, Rab22a overexpression resulted in down-regulation of E-cadherin and up-regulation of N-cadherin in OC cells, indicating a novel mechanism underlying migration and invasion of EOC cells.

In summary, the present study is the first to demonstrate a tumor suppressor role for the miR-373 by targeting Rab22A in human EOC. The miR-373/Rab22a axis provides a novel insight into the pathogenesis of EOC, particularly EOC invasion and metastasis, and represents a new potential therapeutic target for the treatment of this deadly malignancy.

## MATERIALS AND METHODS

### Cell lines and human tissue samples

The human epithelial ovarian cancer (EOC) cells (SKOV3, A2780, and CP70) were purchased from the American Type Culture Collection (ATCC, Manassas, VA) and were cultured in RPMI-1640 (Gibco, Foster City, CA) supplemented with 10% (w/v) fetal bovine serum (FBS; Gibco) and 1% penicillin-streptomycin (Gibco). Human ovarian immortalized nontumorigenic human ovarian surface epithelial (IOSE) and EOC cells (HeyC2) that were originally obtained from the ATCC were gifts from Prof. MW-Y Chan (National Chung Cheng University, Taiwan). Human embryonic kidney 293T (HEK293T) cells were cultured in Dulbecco's Modified Eagle's Medium (Gibco) with 10% FBS and 1% penicillin-streptomycin. All cell lines were maintained at 37°C in a humidified atmosphere with 5% CO_2_. Cell line authentication was performed according to UKCCCR Guidelines every 3–4 months, including mycoplasma detection, DNA –Fingerprinting, isozyme detection and cell vitality detection.

A total of 45 tissue specimens, including 30 primary epithelial ovarian cancer (PEOC) tissues and 15 benign ovarian epithelial tumor tissues, were collected from patients who underwent surgery at department of Obstetrics and Gynecology, Ren-Ji Hospital, Shanghai Jiao Tong University School of Medicine, Shanghai, China. All specimens were snap frozen on collection within 1 hour of surgery at −80°C and frozen sections were cut and mounted on slides. The slides were stained with H&E and examined by a pathologist to ensure >85% presence of tumor cells. Among the 30 PEOC tissue specimens, 10 tumors were determined to be stage I–II and 20 tumors were determined to be stage III–IV high grade serous ovarian cancer. All samples were collected at surgery and prior to chemotherapy. Written informed consent was obtained from each patient, and the use of clinical specimens was approved by the Institutional Ethics Committee.

### RNA extraction and quantitative real-time RT-PCR

Total RNA was extracted using TRIzol reagent (Applied Biosystems, Foster City, CA). Complementary DNA was synthesized with Reverse Transcription kit (Applied Biosystems). Quantitative reverse transcription-polymerase chain reaction (RT-PCR) analyses were performed in triplicate with the SYBR® Green PCR Master Mix (TaKaRa, Otsu, Shiga, Japan) and glyceraldehyde 3-phosphate dehydrogenase (GAPDH) was used as an internal control. The data analysis was performed using the 2^−ΔΔCT^ method as ΔΔCT = (CT_Target1_-CT_GAPDH_) - (CT_Target2_-CT_GAPDH_).

The miRNA fraction in tumor tissues and cells was extracted with miRNA isolation kit (Applied Biosystems). MiRNA-specific reverse transcription was performed with TaqMan^®^ MicroRNA Reverse Transcription Kit (Applied Biosystems). Quantitative RT-PCR was performed using TaqMan^®^ Universal PCR Master Mix (Applied Biosystems) on an ABI 7300HT real-time PCR system (Applied Biosystems), and U6 small nuclear RNA was used as an internal control.

### Plasmid construction

The pCMV6-Rab22a vector and negative control pCMV6-NC vector were amplified from OriGene (Rockville, MD). The short hairpin RNA interfering vector (pLenti-shRab22a) and nontarget control vector (pLenti-NC) were purchased from Thermo Scientific (South Logan, UT). The human Pre-miR-373 sequence was amplified from normal human genomic DNA and cloned into pMSCV vector to generate pMSCV-miR-373.

In the luciferase reporter vector, the wild-type or mutant 3′-UTR of Rab22a was cloned into the downstream of the renilla luciferase gene in the psiCHECK2 vector (Promega, Madison, WI). Other potential target genes were cloned in a similar manner.

### Transient transfection and stable transfection

Rab22a siRNAs were purchased from GenePharma (Shanghai, China). Anti-miR-373 inhibitor (anti-miR-373) and negative control (anti-miR-NC) were purchased from Ambion (Life Technologies, Foster City, CA). Cells were transiently transfected with oligo nucleic acid or vectors using Lipofectamine 2000 (Invitrogen, Carlsbad, CA) and were used 48 h posttransfection.

Virus particles were harvested from HEK293T cells 48 h after transfection with pMSCV-miR-373 or pLenti-shRab22a with the packaging plasmids using Lipofectamine 2000. SKOV3 cells were infected with virus supernatant fluid with 8 μg/mL polybrene and selected in puromycin for 72 h at 1.5 mg/mL.

### Luciferase assay

After 48 h of transfection, cells were lysed with 1× reporter lysis buffer and firefly and renilla luciferase activities were measured using the Dual-Luciferase® Reporter kit (Promega) according to the manufacturer's instructions. Firefly luciferase activity was standardized to the renilla activity as a control.

### *In vitro* migration, invasion, and wound scratch assays

SKOV3 and A2780 cells were transfected with miR-373 (negative control precursor) or Rab22a siRNA (or negative control precursor). For the transwell invasion assay, 1 × 10^5^ cells per chamber for SKOV3 and 1.5 × 10^5^ cells per chamber for A2780 were plated (BD Biosciences, Franklin Lakes, NJ). Cells were allowed to invade through the matrigel-coated inserts at 37°C for 16 h (SKOV3 cells) or 30 h (A2780 cells). For the migration assays, 5 × 10^4^ SKOV3 cells or A2780 cells were plated into each chamber without matrigel for 12 h. Cell migration and invasion were determined by counting five random fields at high-power (Olympus Corp., Tokyo, Japan).

For the wound scratch assay, 5 × 10^6^ SKOV3 cells that stably overexpressed either miR-373 or empty vector were plated and grown until confluent state and then cells were scratched using sterile tips. Cellular migration (toward the scratched area) was assessed after 24 h.

### *In vivo* metastasis assays

Female BALB/c nu/nu mice aged 6 weeks were kept under pathogen-free conditions according to Shanghai Medical Experimental Animal Care guidelines. Animal protocols were approved by the Institutional Animal Care and Use Committee of Shanghai JiaoTong University School of Medicine. Ovarian cancer SKOV3 cells stably transfected with the Luc gene (SKOV3^luc^) were generated. For the *in vivo* metastasis assays, eight mice in each group were injected i.p. with 5 × 10^6^ SKOV3^luc^-pMSCV-miR-373 cells in 200 μL PBS and SKOV3^luc^-pMSCV-NC cells as control, respectively. Each week all mice were anesthetized and given an i.p. injection of D-luciferin (GoldBio Technology, St. Louis, MO) and 10–15 min after the injection, bioluminescence images were captured with IVIS Spectrum (Xenogen, Shrewsbury, MA). Five weeks after i.p. injection, the mice were killed and the number of visible tumors in the cavity was counted.

### Western blotting

For the protein expression analyses, cells were lysed with 1 × SDS. Proteins were separated on 8-15% SDS-polyacrylamide gel electrophoresis and transferred onto polyvinylidene difluoride membranes (Millipore, Billerica, MA). Immunoblots were performed with primary monoclonal antibodies Rab22a (Abcam, Cambridge, UK), E-cadherin (R&D Systems, Minneapolis, MN), N-cadherin (BD Biosciences) and β-actin antibodies (Sigma-Aldrich, St. Louis, MO). Later the blots were incubated with horseradish peroxidase-linked goat-anti-mouse or goat-anti-rabbit secondary antibody (Abcam). The signals were detected by ChemiLucent ECL Detection system (Millipore). The results of Western blots were analyzed by the Image J program.

### Immunohistochemical staining

Immunohistochemical (IHC) staining was performed on 5-μm sections of paraffin-embedded human EOC tissues and benign ovarian tumor tissue to determine the expression of Rab22a using Rab22a antibody (Abcam). Briefly, the slides were incubated in 1 mg/mL Rab22a antibody (1:50) overnight. The sections were then incubated with biotinylated anti-rabbit antibody for 1 h followed by treatment with 3,3′-diaminobenzidine (Sigma-Aldrich) working solution and counterstaining with hematoxylin. Scoring was based on the proportion of positively stained cells: <5% was scored as 0; 5–24% was scored as 1; 25–49% was scored as 2; 50–74% was scored as 3; and >74% was scored as 4.

### Microarray analysis

The SKOV3 cells were transfected with pMSCV-NC vector and pMSCV-miR-373 as described above. Total RNA was isolated and labeled. The 35k human Genome array (CapitalBio, Beijing, China) was used for gene expression analysis. The microarray data were were analysed using SpotDataT Pro V3.0. Differentially expressed genes were identified through the fold change set at <0.5-fold (down-regulated) or >2 (up-regulated) change. Microarray analysis was performed using one biological replicate.

### Statistical analysis

All the experiments were carried out at least in triplicate. The data were presented as mean ± standard deviation. The differences between two groups were analyzed using the double-sided Student's t-test. The statistical analyses of cases in groups were performed with the Chi-square test. *P* < 0.05 indicated a statistically significant difference.

## SUPPLEMENTARY FIGURE AND TABLE


