# Supplementary material for: MiR-373 targeting of the Rab22a oncogene suppresses tumor invasion and metastasis in ovarian cancer
Source: Oncotarget. 2014 Nov 7;5(23):12291–303. doi: 10.18632/oncotarget.2577 (PMC4323008; doi:10.18632/oncotarget.2577)
Supplement: Supplementary file 1 [file oncotarget-05-12291-s001.pdf]

SUPPLEMENTARY FIGURE AND TABLE

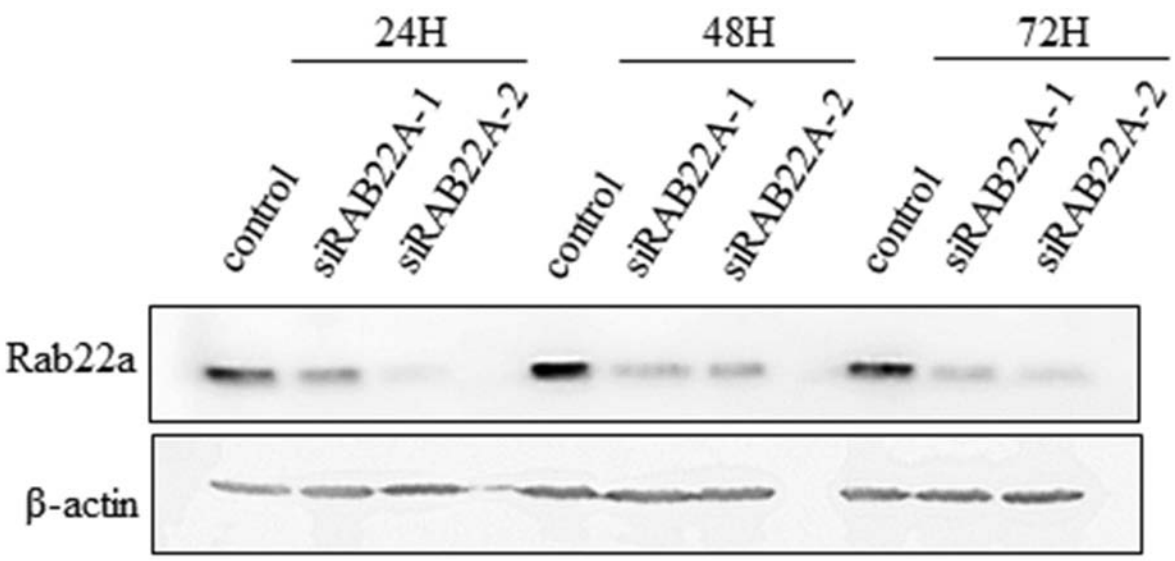

Supplementary Figure S1: siRab22a-2 inhibited Rab22a protein more efficiently than siRab22a-1.

Supplementary Table S1. SKOV3-miR-373

SKOV3-pMSCV-NC	SKOV3-pMSCV-miR-373	Ratio	Name	Description
2615	120	0.0336	TNC	Tenascin-C
857	120	0.1156	IL6	Interleukin-6
1212	190	0.1331	IL7R	interleukin 7 receptor
1739	389	0.1621	MPP1	membrane protein, palmitoylated 1
8514	1202	0.1653	TGFBI	transforming growth factor, beta-induced,
504	98	0.1781	SPINK6	Serine protease inhibitor Kazal-type 6
855	216	0.1818	SNAI2	Zinc finger protein SLUG
3198	663	0.1826	ALDH2	aldehyde dehydrogenase 2 family (mitochondrial)
11712	3130	0.1892	CTSB	Cathepsin B
418	-5	0.1998	SERPINB5	Serpin B5
625	138	0.2162	VPREB3	Pre-B lymphocyte protein 3
565	164	0.2209	FAM50B	Protein FAM50B
941	262	0.2239	IFI44L	interferon-induced protein 44-like
1773	504	0.2286	F8	Coagulation factor 8
2808	762	0.2382	ELAVL2	ELAV-like protein 2
2808	762	0.2382	S100A2	S100 calcium binding protein A2
4437	899	0.2385	LOX	lysyl oxidase
1093	291	0.2407	CPM	Carboxypeptidase M
486	192	0.2419	SPANXE	SPANX family, member E
879	263	0.2452	CHRNA9	Neuronal acetylcholine receptor subunit alpha-9
560	172	0.2503	PRKCH	protein kinase C, eta
856	242	0.2576	JUN	jun proto-oncogene
415	124	0.2579	SART2	dermatan sulfate epimerase
1827	666	0.2818	CLIC3	Chloride intracellular channel protein 3
1377	483	0.2829	PSB9	Proteasome subunit beta type 9
673	171	0.2841	TMOD1	Tropomodulin-1
615	224	0.2853	SOAT1	Sterol O-acyltransferase 1
526	204	0.2949	UTRN	Utrophin
1015	253	0.2952	CPT1C	"Carnitine O-palmitoyltransferase I, brain isoform
4671	1794	0.3033	EMD	Emerin
1777	755	0.3035	F8A2	Factor VIII intron 22 protein
716	226	0.3052	TPST1	Protein-tyrosine sulfotransferase 1
1529	584	0.3086	TRIO	trio Rho guanine nucleotide exchange factor

(*Continued*)

SKOV3-pMSCV-NC	SKOV3-pMSCV-miR-373	Ratio	Name	Description
2163	861	0.3089	TNNT1	troponin T type 1 (skeletal, slow)
6041	2129	0.3111	SERPINE1	serpin peptidase inhibitor, clade E (nexin, plasminogen activator inhibitor type 1), member 1
9182	2771	0.317	IFI27	interferon, alpha-inducible protein 27
5824	2367	0.3201	ASPH	Aspartyl/asparaginyl beta-hydroxylase
2864	838	0.3213	FAM105A	family with sequence similarity 105, member A
1631	624	0.3216	FAM114A1	family with sequence similarity 114, member A1
4143	1930	0.323	SOD2	superoxide dismutase 2, mitochondrial
1300	520	0.3246	AKAP12	A-kinase anchor protein 12
2604	1047	0.3274	FN1	Fibronectin 1
1195	504	0.3294	CETN2	Centrin-2 (Caltractin isoform 1)
5410	1909	0.3302	CA12	Carbonic anhydrase 12
773	255	0.3377	ROM1	retinal outer segment membrane protein 1
2749	1079	0.3387	NPC1	Niemann-Pick type C1
704	255	0.3412	SEC24D	Protein transport protein Sec24D
2163	790	0.3489	CD24	Signal transducer CD24
3788	1942	0.351	FAM50A	family with sequence similarity 50, member A
569	274	0.3517	ST5	Suppression of tumorigenicity 5
6029	2470	0.3555	FOLR1	folate receptor 1
2070	702	0.3564	UBE2L6	Ubiquitin/ISG15-conjugating enzyme E2 L6
1161	436	0.3626	DDX10	DEAD (Asp-Glu-Ala-Asp) box polypeptide 10
7938	4238	0.3643	CALU	Calumenin
472	222	0.3682	TRO	Trophinin
5814	1627	0.3692	CFAB	Complement factor B
6041	2822	0.3709	TBL2	transducin (beta)-like 2
6676	3717	0.3738	ADM	adrenomedullin
20000	9292	0.3796	TM4SF1	Transmembrane 4 L6 family member 1
446	198	0.3803	ANG	angiogenin, ribonuclease, RNase A family, 5
7927	3371	0.3804	CTSL	Cathepsin L
5916	3272	0.3845	HLA-H	major histocompatibility complex, class I, H
1143	407	0.386	WFDC3	WAP four-disulfide core domain 3

(*Continued*)

SKOV3-pMSCV-NC	SKOV3-pMSCV-miR-373	Ratio	Name	Description
569	300	0.3889	TCP11L1	t-complex 11 (mouse) like 1
826	262	0.3907	PDGFRL	platelet-derived growth factor receptor-like
2159	948	0.3919	DIO2	Type II iodothyronine deiodinase
1866	877	0.3932	SPHK1	Sphingosine kinase 1
4453	1635	0.3945	LAGE3	L antigen family member 3
1200	376	0.3965	CPZ	Carboxypeptidase Z
1228	651	0.3986	LMO1	LIM domain only 1 (rhombotin 1)
1003	459	0.4046	CDC42	Cell division control protein 42 homolog
10291	5340	0.406	VDAC1	voltage-dependent anion channel 1
1333	755	0.4107	IL6ST	interleukin 6 signal transducer
1342	737	0.4114	LAMA4	Laminin subunit alpha-4
10659	6047	0.4121	LTB4DH	ltb4dh protein
6478	3888	0.4134	CMAS	N-acylneuraminate cytidylyltransferase
489	223	0.4138	TMEM41B	transmembrane protein 41B
2309	971	0.4187	RNF24	ring finger protein 24
1042	408	0.4202	P4HA2	prolyl 4-hydroxylase, alpha polypeptide II
1626	539	0.4204	KDELR3	KDEL (Lys-Asp-Glu-Leu) endoplasmic reticulum protein retention receptor 3
1646	669	0.4205	PCOLCE2	Procollagen C-endopeptidase enhancer 2
7116	4068	0.4211	HLA-B	major histocompatibility complex, class I, B
2096	1092	0.4218	CXCR4	C-X-C chemokine receptor type 4
471	242	0.4225	FAM18B	Protein FAM18B
2538	1180	0.4231	FBXO2	F-box only protein 2
4985	2467	0.4242	SERPINE2	serpin peptidase inhibitor, clade E (nexin, plasminogen activator inhibitor type 1), member 2
1219	626	0.4244	RAB32	RAB32, member RAS oncogene family
1119	629	0.4288	MYO1D	myosin ID
730	344	0.4303	UACA	Uveal autoantigen with coiled-coil domains and ankyrin repeats protein
1270	601	0.4307	SLC10A3	solute carrier family 10, member 3
9346	5619	0.4342	THEM2	Thioesterase superfamily member 2
554	245	0.4359	SPANXB2	SPANX family, member B2
864	487	0.4364	ADAM12	A disintegrin and metalloproteinase domain 12
3154	1712	0.4378	IFI16	interferon, alpha-inducible protein 16

(*Continued*)

SKOV3-pMSCV-NC	SKOV3-pMSCV-miR-373	Ratio	Name	Description
8938	4775	0.4386	ERO1L	ERO1-like protein alpha
1501	859	0.4406	MBOAT5	Membrane-bound O-acyltransferase domain-containing protein 5
3138	1329	0.4425	HLA-C	major histocompatibility complex, class I, C
1608	967	0.4447	SNX10	Sorting nexin-10
1345	723	0.4454	LRP10	Low-density lipoprotein receptor-related protein 10
1552	752	0.4483	SH3KBP1	SH3 domain-containing kinase-binding protein 1
1265	575	0.4494	AAMDC	adipogenesis associated, Mth938 domain containing
871	489	0.4502	TMEM30A	transmembrane protein 30A
5084	3005	0.4518	DERA	Putative deoxyribose-phosphate aldolase
3089	1455	0.4546	NSDHL	NAD(P) dependent steroid dehydrogenase-like
1896	1062	0.4548	ACBD3	acyl-CoA binding domain containing 3
1421	633	0.4557	PLOD2	Procollagen-lysine,2-oxoglutarate 5-dioxygenase 2
1319	705	0.4571	ANKRD18B	ankyrin repeat domain 18B
3835	2412	0.4577	CEECAM1	cerebral endothelial cell adhesion molecule 1
5821	3776	0.4584	M6PRBP1	Mannose-6-phosphate receptor-binding protein 1
4869	2408	0.4588	IGFBP3	Insulin-like growth factor-binding protein 3
1370	636	0.4591	PMP22	Peripheral myelin protein 22
4324	2218	0.4609	QSCN6	Sulfhydryl oxidase 1
3669	1978	0.461	AGPS	Alkylglycerone-phosphate synthase
679	403	0.4616	PDIA5	Protein disulfide-isomerase A5
851	508	0.462	CD44	CD44 antigen precursor
4608	2253	0.4623	PALM2	paralemmin 2
454	244	0.4634	TSPAN8	Tetraspanin-8
2889	1392	0.4644	PRKCDBP	protein kinase C, delta binding protein
3883	2300	0.465	SNRP70	small nuclear ribonucleoprotein 70 kDa (U1)
504	275	0.4663	TBC1D10A	TBC1 domain family member 10A
739	400	0.4673	PRRC1	proline-rich coiled-coil 1
3259	1729	0.4674	DKC1	H/ACA ribonucleoprotein complex subunit 4

(*Continued*)

SKOV3-pMSCV-NC	SKOV3-pMSCV-miR-373	Ratio	Name	Description
4364	2571	0.4674	RABAC1	Rab acceptor 1 (prenylated)
1481	926	0.4691	CAMK2N1	calcium/calmodulin-dependent protein kinase II inhibitor 1
5414	2550	0.4698	LARP6	acheron isoform 1
2222	1008	0.4699	COL5A1	Collagen alpha-1(V) chain precursor
3360	1816	0.4711	RND3	Rho family GTPase 3
5515	2682	0.4713	MARCH6	membrane-associated ring finger (C3HC4) 6
3248	1496	0.4714	ENO2	enolase 2 (gamma, neuronal)
1248	609	0.4715	DTWD1	DTW domain containing 1
4052	2283	0.4717	PFN2	Profilin-2
3985	1532	0.4719	IDH2	isocitrate dehydrogenase 2 (NADP+), mitochondrial
5958	3864	0.4753	HIST1H2AA	histone cluster 1, H2aa
4194	2591	0.4778	HMOX1	Heme oxygenase 1
30	11	0.4806	GALNT3	Polypeptide N-acetylgalactosaminyltransferase 3
1058	653	0.4834	GLIPR1	Glioma pathogenesis-related protein 1
1090	608	0.4838	AYTL1	Acyltransferase-like 1
4564	2815	0.484	CAPZA1	F-actin capping protein subunit alpha-1
1233	778	0.4845	MR1	major histocompatibility complex, class I-related
1975	1069	0.4846	MSH6	mutS homolog 6
6513	3882	0.4851	GOLPH3	Golgi phosphoprotein 3
2480	1183	0.4852	VDAC4	voltage dependent anion channel 4
803	408	0.4853	MUC1	Mucin-1
1201	621	0.4858	SYBL1	Synaptobrevin-like protein 1
2260	1255	0.486	RPL22L1	Ribosomal protein L22-like 1
51	17	0.4879	ANK2	Ankyrin-2
1267	892	0.4895	RAB22A	RAB22A, member RAS oncogene family
2913	1383	0.4902	NDRG1	N-myc downstream regulated 1
1667	793	0.4911	RRAGA	Ras-related GTP-binding protein A
567	312	0.4917	TRIP11	Thyroid receptor-interacting protein 11
2440	1241	0.4933	CCDC77	Coiled-coil domain-containing protein 77
4313	2426	0.4935	SURF4	Surfeit locus protein 4
2006	1081	0.4947	FLNA	Filamin-A
2369	1303	0.4947	PSMC2	Proteasome 26S subunit ATPase 2
4140	2189	0.4955	FASN	Fatty acid synthase

(*Continued*)

SKOV3-pMSCV-NC	SKOV3-pMSCV-miR-373	Ratio	Name	Description
4048	2383	0.4956	IGFBP6	Insulin-like growth factor-binding protein 6
2731	1538	0.4956	POPDC3	Popeye domain-containing protein 3
4865	3321	0.4958	EIF4A2	eukaryotic translation initiation factor 4A2
17675	11450	0.4961	ARF1	ADP-ribosylation factor 1
20329	13286	0.4961	RPL10	60S ribosomal protein L10
452	270	0.4965	HRSP12	heat-responsive protein 12
1135	471	0.4969	LYPD3	Ly6/PLAUR domain-containing protein 3
5366	2753	0.4996	CROT	carnitine O-octanoyltransferase
665	341	0.4996	SGTB	Small glutamine-rich tetratricopeptide repeat-containing protein B
5366	2753	0.4996	VBP1	von Hippel-Lindau binding protein 1
242	485	0.4997	GNPDA2	glucosamine-6-phosphate deaminase 2
299	599	0.4999	SLC38A6	N system amino acid transporter NAT-1
1259	2518	0.5	ARF4	ADP-ribosylation factor 4
